# In Vivo Measurement of Ear Ossicle and Bony Wall Vibration by Sound Stimulation of Cartilage Conduction

**DOI:** 10.3390/audiolres13040044

**Published:** 2023-07-12

**Authors:** Hiroaki Yazama, Shiro Arii, Hideyuki Kataoka, Tasuku Watanabe, Ryo Kamitani, Kazunori Fujiwara

**Affiliations:** 1 Department of Otolaryngology, Head and Neck Surgery, Faculty of Medicine, Tottori University, 36-1 Nishi-machi, Yonago 683-8504, Japan; hkataoka@tottori-u.ac.jp (H.K.); t.watanabe@tottori-u.ac.jp (T.W.); kkamitani-ryo@tottori-u.ac.jp (R.K.); kfujiwa@tottori-u.ac.jp (K.F.); 2 Kanki Rotordynamics Lab, 1646 Higashikanki-cyo, Kakogawa 675-0057, Japan; shiroarii@kyi.biglobe.ne.jp

**Keywords:** cartilage conduction, ossicular vibration, bone vibration

## Abstract

The cartilage-conduction pathway was recently proposed as a third auditory pathway; however, middle-ear vibrations have not yet been investigated in vivo. We aimed to measure the ossicles and bone vibration upon cartilage-conduction stimulation with a non-contact laser Doppler vibrometer. We recruited adult patients with normal ear structures who underwent cochlear implant surgery at our hospital between April 2020 and December 2022. For sound input, a cartilage-conduction transducer, custom-made by RION Corporation (Tokyo, Japan), was fixed to the surface of the tragus and connected to an audiometer to regulate the output. A posterior tympanotomy was performed and a laser beam was directed through the cavity to measure the vibration of the ossicles, cochlear promontory, and posterior wall of the external auditory canal. Five participants (three men, mean age: 56.4 years) were included. The mean hearing loss on the operative side was 96.3 dB HL in one patient, and that of the other patients was off-scale. The vibrations were measured at a sound input of 1 kHz and 60 dB. We observed vibrations of all three structures, demonstrating the existence of cartilage-conduction pathways in vivo. These results may help uncover the mechanisms of the cartilage-conduction pathway in the future.

## 1. Introduction

**Figure 1:**
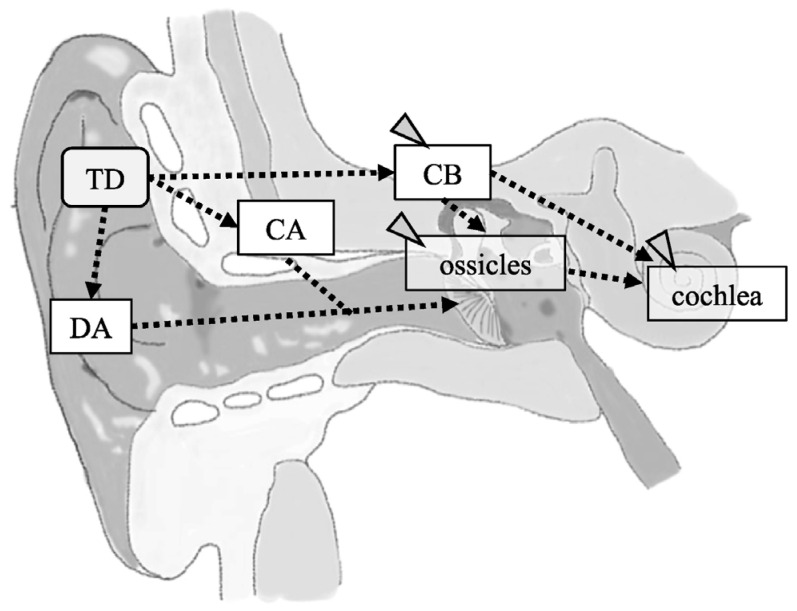
A schema of the structures contributing to cartilage conduction (CC) pathways. CC is achieved via a direct air-conducted pathway (DA), cartilage–bone-conducted pathway (CB), and cartilage–air-conducted pathway (CA). Dashed lines indicate predicted pathways. The gray arrowheads indicate the pathway and structures to be analyzed in this study. TD, cartilage-conducting transducer.

Sound has conventionally been thought to be transmitted through two pathways: air conduction and bone conduction. In air conduction, vibrations in the air are transmitted to the tympanic membrane, where they are converted into mechanical vibrations that amplify the sound pressure as they travel through the ossicles to the cochlea. Bone conduction mainly induces mechanical vibrations in the temporal bone and skull, which are subsequently transmitted to the cochlea. However, bone conduction may occur through multiple pathways, including through the cerebrospinal fluid and ossicles. The sound transmission mechanisms for these pathways have been extensively investigated and are clearly explained by Stenfelt et al. [1]. Recently, Hosoi et al. [2] proposed cartilage conduction as a third auditory pathway. They showed that sound generated by a cartilage-conduction transducer usually reaches the inner ear via three different pathways in humans with normal anatomical structures: the direct air-conduction, cartilage–air-conduction, and cartilage–bone-conduction pathways (Figure 1). In direct air-conduction, sound is transmitted to the cochlea via conventional air conduction. In cartilage–air-conduction, vibrations of the auricular cartilage induce acoustic signals in the ear canal, which are transmitted to the cochlea via conventional air conduction. In cartilage–bone-conduction, vibrations from the auricular cartilage are transmitted to the cochlea via the temporal bone. The acoustic estimation of these conduction pathways has been reported by Nishimura et al. [3] and Shimokura et al. [4]. Nishimura et al. [5] investigated which pathway is dominant for cartilage conduction, concluding that it is the cartilage–air-conduction pathway. However, evidence for the existence of the two cartilage-conduction pathways, cartilage–air-conduction and cartilage–bone-conduction, is currently insufficient in terms of whether the vibrations are actually being transmitted along them. Although such evidence has been produced in a model of the external auditory canal [6], in vivo validation in humans is lacking. Therefore, measurement of the vibration of the ossicles during cartilage conduction in vivo may provide useful information.

We previously analyzed vibrations in the human tympanic membrane and ear ossicles induced by acoustic excitation using a non-contact laser Doppler vibrometer (LDV) and examined how sound pressure acting on the tympanic membrane is transmitted to the cochlea through the middle-ear sound-transduction system [7,8]. In particular, we focused on the phase difference and amplitude of the measured signal relative to the excitation signal to evaluate the state of ossicular vibration. In this study, we attempted to demonstrate the existence of all three pathways of cartilage conduction using the same method as previously reported to measure the vibrations of the ossicles, cochlear promontory, and bones of the external auditory canal by using a cartilage-conduction transducer. Such measurements have not been performed in humans with an almost physiologically intact middle-ear conduction system, as in the present study. In this study, we aimed to confirm the presence of the cartilage-conduction pathway in vivo and to evaluate how much of the transmitting force is transmitted to the ossicles and bones. Moreover, the dominant pathway is the cartilage–air-conduction pathway, and measurements of ossicular vibration transmitted via cartilage conduction should yield results similar to those transmitted via tympanic membrane vibration. Therefore, we also compared these measurements with our previously reported measurements of ossicular vibration via the air-conduction pathway.

## 2. Materials and Methods

### 2.1. Participants

In this study, participants were recruited from patients who underwent cochlear implant surgery at our hospital between April 2020 and December 2022. We selected participants with normal structures of the external, middle, and inner ear to minimize errors in measuring the vibration of the ossicles, cochlear promontory, and external auditory canal wall. In addition, we selected patients in whom the middle ear was fully developed. Therefore, the selection criteria were as follows: at least 20 years of age at the time consent was obtained; no external or middle ear disease; no malformation of the ossicles or inner ear; surgery to open the middle ear cavity was planned; and consent was obtained from the patients. As the only patients who met these criteria were patients with cochlear implants, we included adult patients undergoing cochlear implant surgery. The exclusion criteria were a lack of consent or withdrawal of consent for participation in the study.

This study was approved by the Tottori University Ethics Review Committee (approval number: 2100). All the participants were informed of the research aims, and their written consent was obtained before their inclusion in the study.

### 2.2. Output Characteristics of the Cartilage-Conduction Transducer

The output characteristics of the cartilage-conduction transducer were measured to determine how much vibration was induced by the force generated. These measurements were performed by RION Corporation (Tokyo, Japan), the developer of the transducer. They used an artificial mastoid (Artificial Mastoid, B&K 4930; Brüel & Kjær, Nærum, Denmark) for the measurements. A cartilage-conduction transducer was connected to an audiometer (RION AA-73A; RION Corporation, Kokubunji, Japan), and the excitation and output characteristics were measured, the results of which were provided to us.

### 2.3. Vibration Generation and Vibration Measurement Equipment

**Figure 2:**
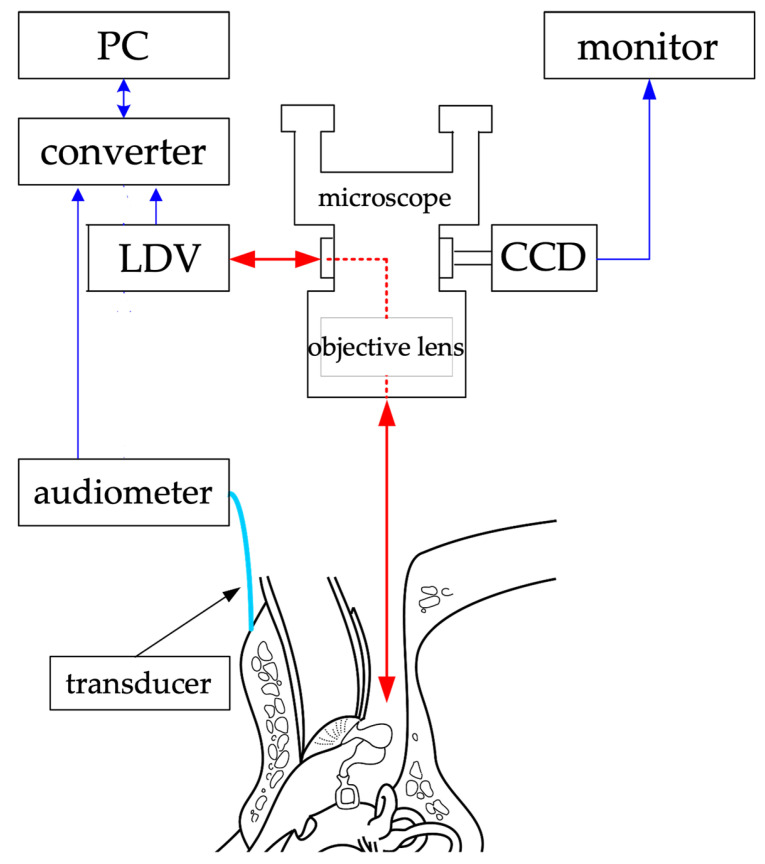
Experimental system for measurement of vibration. The red arrows represent the incoming and outgoing laser beams, and the blue arrows represent the transmission and reception of data. The arrowheads indicate the direction of data and laser exchange. Laser beams are emitted through a microscope to measure vibrations at various points. PC: personal computer, LDV: laser Doppler vibrometer, CCD: charge-coupled device camera. (Reproduced from Kunimoto et al. [8], with permission.)

The cartilage-conduction transducer, the source of the vibrations used in this study, was custom-made by RION Corporation (model number: F0198L1). It was connected to an audiometer (RION AA-73A) for the ability to adjust the sound output. Figure 2 is a schema of the experimental system for vibration measurement. In the system, a surgical microscope (OPMI; Zeiss, Oberkochen, Germany) is usually equipped with an eyepiece and a CCD camera located between the objective and the eyepiece. Instead of an eyepiece, an LDV (VH300; Ometron, Hertfordshire, UK) was mounted, using a goniometer to adjust the laser beam and the visual axis. The laser beam and microscope focus were adjusted before the measurements were taken. As a result, the laser beam was bent by the prism of the eyepiece along the visual axis of the microscope and delivered through the objective lens to the measurement site. The laser beam was reflected from the measurement site back to the LDV.

The LDV operates by comparing the frequency of an emitted beam with that of the beam reflected from a moving surface. The accuracy of the comparison between the emitted and reflected beams depends on the amplitude of the reflected beam that returns to the velocity decoder. Clearly delineated amplitudes were extracted because too small an amplitude would result in noisy velocity estimates. The laser output power was adjusted to less than 1 mW in accordance with the safety standards of the U.S. Food and Drug Administration. The measured data were recorded and digitized using an analog-to-digital converter (PULSE356-B-130; Brüel & Kjær) with a sampling frequency of 131,072 (=2^17^) Hz. The vibration frequency component of the cartilage-conduction transducer was extracted from the measured velocity signal by using a lock-in amplifier algorithm, and the vibration amplitude was obtained by integrating the velocity at the frequency of the excitation signal. The phase difference of the excitation signal was also obtained.

### 2.4. Vibration Measurement

**Figure 3:**
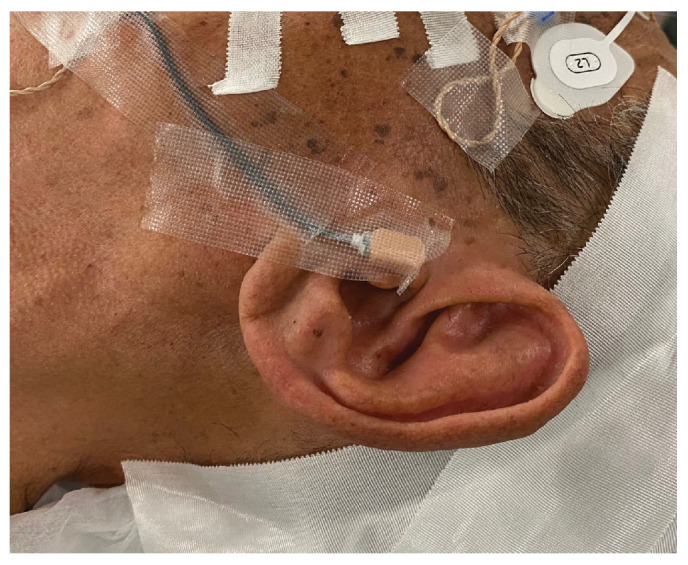
Fixed transducer. The transducer is attached to the surface of the tragus by using double-sided tape and covered with waterproof tape to secure it in place.

**Figure 4:**
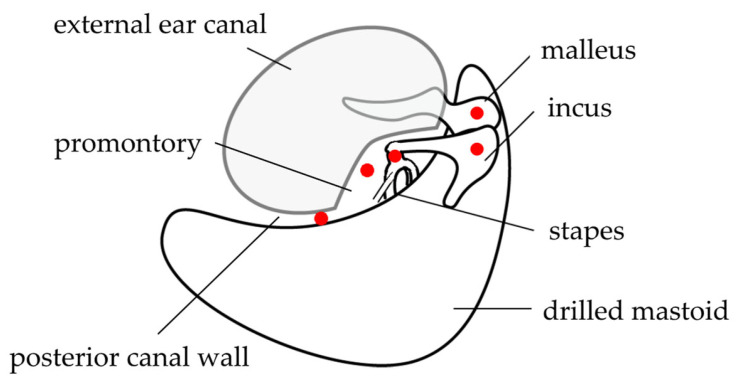
Measurement points. Mastoidectomy and posterior tympanotomy are performed, and the measurement sites (the malleus head, body of the incus, incudostapedial joint, cochlear promontory, and the mastoid side of the posterior wall of the external auditory canal after mastoidectomy) are placed under clear view. This figure was modified from Kunimoto et al. [8], with permission.

For the sound pressure input, a transducer was fixed to the skin surface of the tragus with double-sided tape, covered with waterproof tape, and disinfected (Figure 3). After a mastoidectomy under general anesthesia without muscle relaxants, a posterior tympanotomy was performed, and the round window niche and superstructure of the stapes were identified. The operating and measuring microscopes were exchanged while maintaining a clean field. A laser beam was produced by the LDV and directed through the cavity. The focus of the laser beam was adjusted according to the monitor. The measurement sites were the malleus head, incus body, incudostapedial (I-S) joint, cochlear promontory, and posterior wall of the external auditory canal (Figure 4). The audiometer was set to an output of 1 kHz at 60 dB, and the velocity and phase were measured at each measurement site. The measurements were started at the same time as the tonal stimulus. The measurements at each point took about 5 s. Following the measurements, the microscopes were promptly switched for completion of the operation. We anticipated approximately 30 min of extended anesthesia time for a series of measurement procedures, and none of the participants greatly exceeded the anticipated time.

### 2.5. Vibration Analysis

The relative motion of each ear ossicle was calculated from the measurements in Section 2.4. Continuous amplitude changes at each measurement site were calculated using phase shifts from the sinusoidal excitation. The amplitudes of each measured section were averaged across the measurements and visualized. The accuracy was verified using the same protocol as in a previous report [7].

## 3. Results

### 3.1. Participant Characteristics

Nineteen patients underwent cochlear implant surgery at our institution between April 2020 and December 2022. Among these, 10 patients were excluded because they were under 20 years of age, and one adult patient was excluded because of an inner-ear malformation (please see the inclusion and exclusion criteria described in the Section 2). Consent for participation was obtained from six of the eight remaining patients. One of these participants was excluded from the analysis because of poorly recorded data. Finally, five participants were included. Their mean age was 56.4 years (range: 42–69), and three were men. The mean hearing loss on the operative side was 96.3 dB HL in one patient, whereas that of the other patients was >100 dB HL.

### 3.2. Output Characteristics of the Cartilage-Conduction Transducer

**Figure 5:**
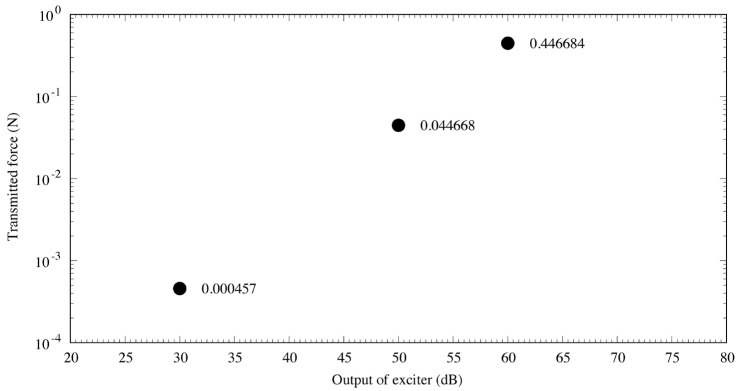
Output characteristics of the cartilage-conduction transducer. The three points represent the transmitted force from the cartilage–conduction transducer corresponding to a certain audiometer dial setting, and the value next to each point is the measured value (N). The output results demonstrate linearity with the transmitted force, indicating that the transducer performed very well.

The measurement results are displayed in Figure 5. The output of the cartilage-conducting transducer was very strong: the transmission force used in the experiment was 446,684 μN, at a frequency of 1 kHz and audiometer output of 60 dB. Assuming a tympanic membrane diameter of 1 cm and sound pressure of 100 dB SPL (2 × 10^6^ μPa), the input from the tympanic membrane to the ossicles was 157 μN, which is approximately 2800 times greater than that with acoustic excitation at 100 dB SPL [7].

### 3.3. Vibration Measurement

**Figure 6:**
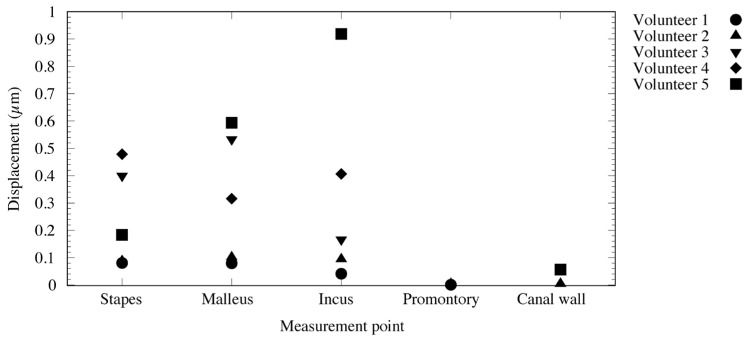
State of ossicle vibration during cartilage-conducted stimulation.

**Figure 7:**
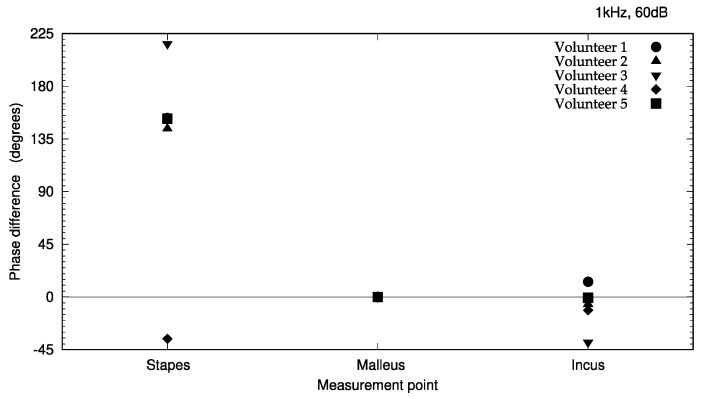
Phase differences of ossicle vibration relative to excitation signal during cartilage–conducted stimulation. The phase of the incus and the stapes with respect to the malleus is indicated. The phase at each measurement point is expressed as the phase difference compared to the reference phase.

The measured vibration responses are listed in Table 1 and illustrated in Figure 6 and Figure 7. At a vibration frequency of 1 kHz and an audiometer output of 60 dB, we were able to measure the vibrations of the I-S joint, malleus head, and body of the incus for all the participants. The smallest vibration amplitude was 0.04 μm and the largest was 0.9 μm. The phase difference in the response to the excitation force indicates that the malleus head and body of the incus vibrate in almost the same phase. The I-S joint and malleus head vibrate in nearly opposite phases, with the exception of those in participant 4. Vibrations of the cochlear promontory could only be measured in participants 1 and 2. These amplitudes were very small compared to those of the ossicles (on the order of 1/100). The vibrations of the posterior wall of the external auditory canal could be measured in participants 2 and 5. Again, these amplitudes were very small compared to those of the ossicles, on the order of 1/100 for participant 1 and 1/10 for participant 2.

## 4. Discussion

Vibrations generated in the ear ossicles or bones indicate the transmission of a force, such as sound pressure. An evaluation criterion is needed to compare the state of transmission among different pathways. In air-conducted vibration, the excitation force transmitted to the ossicles can be estimated from the sound pressure input from the tympanic membrane [7]. On the other hand, we measured the force produced by the cartilage-conduction transducer as the force transmitted to the site where the transducer was attached; the actual force acting on the ossicles cannot be estimated. We believed that the magnitude of the vibration of the ossicles during air-conducted vibration could be used as a crude criterion for the transmitted force, indicating a large or small force. Therefore, we focused on the vibration state, especially the vibration amplitude, in this study.

LDV is a noncontact optical technique used for basic research on the dynamics of hearing [9,10,11]. Such studies have been conducted on the temporal bones of live humans and those of cadavers [9,10,12,13,14,15]. We previously reported measuring the vibrations of the ossicles and tympanic membrane in response to acoustic stimulation via the air-conducted pathway [7,8]. In the present study, we applied the same method to measure the vibration of the ossicles, external auditory canal bone, and cochlear promontory in response to excitation from a cartilage-conduction transducer and attempted to verify the cartilage-conducted pathway. We believe that LDV is the most appropriate measurement method for two reasons. First, contact-type vibration measuring devices may be affected by the dead weight of the transducer itself, which may suppress fine vibrations. Second, as the measurements were to be made within the surgical field, sterility was crucial.

The measurement results (Table 1) appear to reveal interindividual differences in amplitude. Two explanations for these differences may be provided. First, the difference in size and shape of the auricular cartilage between the individuals might have resulted in differences in the degree of adhesion of the transducer. In fact, the conduction efficiency changes just by shifting the location of the transducer [2]. Second, differences in the angle of incidence of the laser light and the direction of vibration may be considered. The velocity was measured on the axis of the laser beam excitation. Therefore, if the directions of the target vibration and laser excitation do not coincide, only the vibration component of the target in the direction of the laser excitation is measured. In such cases, the value is smaller than the actual vibration component (cosine component). The roughness of and liquid buildup on the surface of the target cause diffusion of the laser-beam reflection, reducing the accuracy of the measurement. As demonstrated in Table 1 and Figure 6 and Figure 7, results that could not be accurately measured were excluded from this study.

In this study, the vibrations could be measured in the stapes, malleus head, and body of the incus in all the subjects. Thus, we have provided evidence that the excitation force from the cartilage-conduction transducer was transmitted to the ossicles via the temporal bone. The maximum amplitude of air-conducted vibration in a previous study was 0.03 μm at 1 kHz and 100 dB output [7], whereas the smallest amplitude was 0.08 μm with cartilage-conducted vibration in this study, and the largest amplitude exceeded 0.5 μm, 17 times larger than that obtained with air-conduction excitation. However, considering that the excitation force of the cartilage-conduction transducer is approximately 2800 times that of the air-conduction excitation, the amplitude produced by cartilage-conduction does not appear to be very large. Although cartilage conduction resulted in greater vibration of the ossicles than air conduction, this pathway has proven to be greatly attenuated during transmission through the temporal bone. The phase difference detected in response to the excitation force (Figure 7) indicates that the vibration state of the ossicles is similar to that of air-conduction transmission [8]. From the vibration pattern, the cartilage-conduction pathway seems to have a similar mechanism of vibration transmission to air conduction. However, given the amplitude, other pathways, such as movement of the ear ossicles, may have an effect. Specifically, the malleus head and the incus body are connected and should have the same phase of vibration. The difference in the phases of the malleus head and incus body in this study (Figure 7) might have been due to changes in the vibrational state during sequential measurements. A linear system would result in the same phase throughout; as this is not the case, the system must contain non-linear elements in various places.

Minute vibrations of the cochlear promontory and posterior wall of the external auditory canal were measured, demonstrating that the excitation force from the cartilage-conduction transducer propagates directly to the bone. However, such vibrations were detected in only two of the five participants. This may be owing to the fact that the vibrations were very weak and therefore susceptible to noise, resulting in poor measurements. Other possibilities are that the cochlear promontory is located in the deepest part of the middle-ear cavity, which is difficult for the laser to reach, and that laser excitation of the posterior wall of the external auditory canal was affected by the technique, such as the difficulty of hitting the wall perpendicularly. On the other hand, in terms of the phase, synchronous vibrations were observed in the stapes, cochlear promontory, and posterior wall of the external auditory canal, respectively, all of which was considered to be almost synchronous with the acoustic vibration. Although bone vibrations were confirmed, several questions remain, such as whether vibrations propagated in the cochlea can be perceived as hearing, and if so, to what extent compared to hearing propagated in the cochlea from otoacoustic vibrations.

Based on the abovementioned questions that remain regarding ossicles and bony vibrations, we discuss the pathways through which vibrations are transmitted to the cochlea via cartilage conduction again.

First, we consider the cartilage–air-conduction pathway, in which the vibrations of the temporal bone are transmitted through the canal wall to the air in the auditory canal, which vibrates the tympanic membrane, similar to the air-conduction pathway. In this study, we demonstrated that the ossicles also vibrated substantially, suggesting that it can also be considered a major transmission pathway.

Second, a possible pathway is the transmission of vibrations from the temporal bone via the surrounding ligaments and tympanic membrane to the ossicles, which transmit to the vibrations to the cochlea. In a broad sense, this pathway is consistent with the cartilage–air-conduction pathway, although the ossicles are unlikely to vibrate and transmit vibrations as efficiently. Further studies are needed to examine the differences between these two cartilage–air-conduction pathways and should include a measurement of the sound pressure in the external auditory canal.

Third is the cartilage–bone-conduction pathway, in which vibrations from the temporal bone are transmitted directly to the cochlea. Although this pathway was investigated by Shimokura et al. [4], they were not able to measure sound pressure in their experiments, possibly because the excitation was measured in the contralateral ear, which might have caused substantial attenuation via a shielding effect. As bone can be considered a viscoelastic material, differences in density, Young’s modulus, and internal damping of various parts of the skull may affect the propagation path of vibrations from the transducer. In this study, the velocity changes in the direction of the laser excitation were below a measurable level in several cases; however, that does not mean that the vibration was not transmitted. The excitation force likely still propagated through the elastic body and could be perceived as hearing. Rather, the fact that the velocity could be measured indicates that the input was reliably propagated. In other words, the fact that bone vibration could be measured is evidence of the cartilage–bone-conduction pathway. In cartilage conduction, the transducer is similar to the voice coil in a speaker and the cartilage itself is thought to have a mechanism similar to that of a speaker diaphragm [2]. As demonstrated in this study, vibration may attenuate as it is transmitted to the bone; thus, transmission may be sufficient to the ipsilateral ear and insufficient to the contralateral ear. If this hypothesis is correct, cartilage-conduction hearing aids may be more effective at localizing sound sources. Further studies on bone conduction in the normal ear are required to determine the mechanism by which vibrations are transmitted, as well as the mechanism by which sound is perceived.

On the other hand, bone microvibrations and excitation forces propagating within the elastic body may have an important role. Stenfelt et al. [16] reported that fluid inertia caused by cochlear vibration had the greatest effect on basal membrane vibration in the normal ear when listening to bone-conducted sound of 0.1–10 kHz. Once that relationship is clarified, the benefits of direct vibration of the cochlear promontory should become apparent. We speculate that if the cochlea itself vibrates, it directly vibrates the organ of Corti without the transmission of vibration from the oval window and directly induces vibrations of the hair cells. This makes sense, as the degree of vibration directly affects the perception of sound loudness. However, the amplitude required to vibrate the organ of Corti is unknown and difficult to determine with fixed specimens or cadavers, because protein denaturation may affect vibration transmission. The amplitude will need to be determined in physiologically intact living organisms.

In the present study, we included only five participants; hence, the results were not averaged and may not be applicable to all adults. Limitations also exist in the interpretation of the data owing to the effects of anatomical differences in the participants, differences in the settings of the measurement equipment, and increased noise due to measurement surface roughness and fluid buildup. In addition, as the participants were different ages, the stiffness of the cartilage and bone was likely not be uniform, which could have caused a sampling bias.

Despite these limitations, the results of this study provide evidence for the mechanism of the cartilage-conduction pathways in vivo. The results of the present study should be explored in more detail in future studies for a better understanding of the vibration-based conduction pathway. For example, research on patients with external auditory canal atresia who undergo middle-ear implant surgery would allow study of the cartilage–soft tissue pathway, which would lead to a more detailed elucidation of the mechanism of cartilage conduction.

## 5. Conclusions

In the present study, we observed vibrations in the ossicles and bones, which provides in vivo evidence for the cartilage–air- and cartilage–bone-conduction pathways. The pattern of ear ossicle vibration induced by cartilage conduction was similar to but much larger than that induced by air conduction. This suggests that the cartilage–air-conduction pathway is not the only significant pathway by which vibrations are transmitted during cartilage conduction. Furthermore, our methodology may be useful for future clarification of the details of vibration transmission patterns.

## Figures and Tables

**Table 1:** Vibratory measurements during cartilage-conducted stimulation.

Participant	Measurement Point	Amplitude (µm)	Phase (Degrees)
Volunteer 1 69 y.o. man	Stapes	0.0806	96.36
	Malleus head	0.0794	249.24
	Incus body	0.0414	236.78
	Promontory	0.0008	135.94
	Canal wall	-	-
Volunteer 2 54 y.o. woman	Stapes	0.0874	149.77
	Malleus head	0.1012	6.10
	Incus body	0.0944	−0.06
	Promontory	0.0040	149.99
	Canal wall	0.0041	153.42
Volunteer 3 42 y.o. woman	Stapes	0.3999	174.64
	Malleus head	0.5338	−41.60
	Incus body	0.1670	−80.34
	Promontory	-	-
	Canal wall	-	-
Volunteer 4 68 y.o. man	Stapes	0.4785	−57.38
	Malleus head	0.3159	−22.72
	Incus body	0.4062	−32.96
	Promontory	-	-
	Canal wall	-	-
Volunteer 5 49 y.o. man	Stapes	0.1833	−67.81
	Malleus head	0.5938	139.81
	Incus body	0.9187	139.11
	Promontory	-	-
	Canal wall	0.0566	−26.25

y.o., years old.

## Data Availability

Data is contained within the article.
